# Alarming multidrug resistance in *Staphylococcus aureus* isolated from raw milk of cows with subclinical mastitis: Antibiotic resistance patterns and occurrence of selected resistance genes

**DOI:** 10.1371/journal.pone.0301200

**Published:** 2024-05-16

**Authors:** Ijaz Ul Haq, Mustafa Kamal, Ayman A. Swelum, Shehryar Khan, Patricio R. De los Ríos-Escalante, Tahir Usman

**Affiliations:** 1 College of Veterinary Sciences and Animal Husbandry, Abdul Wali Khan University Mardan, Mardan Pakistan; 2 Department of Zoology, Abdul Wali Khan University Mardan, Mardan, Pakistan; 3 Department of Animal Production, College of Food and Agriculture Sciences, King Saud University, Riyadh, Saudi Arabia; 4 Department of Biotechnology, Abdul Wali Khan University Mardan, Mardan, Pakistan; 5 Facultad de Recursos Naturales, Departamento de Ciencias Biológicas y Químicas, Universidad Católica de Temuco, Temuco, Chile; 6 Nucleo de Estudios Ambientales UC Temuco, Temuco, Chile; 7 Key Laboratory of Agricultural Animal Genetics and Breeding, National Engineering Laboratory for Animal Breeding, College of Animal Science and Technology, China Agricultural University, Beijing, China; Cornell University College of Veterinary Medicine, UNITED STATES

## Abstract

Bovine mastitis is a widespread and costly disease that affects dairy farming globally, characterized by mammary gland inflammation. Bovine intramammary gland infection has been associated with more than 135 different pathogens of which *Staphylococcus aureus* is the main etiology of sub-clinical mastitis (SCM). The current study was designed to investigate the prevalence, antibiotic resistance pattern, and the presence of antibiotic resistance genes (*mecA*, *tetK*, *aacA-aphD* and *blaZ*) in *S*. *aureus* isolated from the raw milk of cows with subclinical mastitis. A total of 543 milk samples were collected from lactating cows such as Holstein Friesian (n = 79), Sahiwal (n = 175), Cholistani (n = 107), and Red Sindhi (n = 182) from different dairy farms in Pakistan. From the milk samples microscopic slides were prepared and the somatic cell count was assessed to find SCM. To isolate and identify *S*. *aureus*, milk was streaked on mannitol salt agar (MSA) plates. Further confirmation was done based on biochemical assays, including gram staining (+ coccus), catalase test (+), and coagulase test (+). All the biochemically confirmed *S*. *aureus* isolates were molecularly identified using the thermonuclease (*nuc*) gene. The antibiotic resistance pattern of all the *S*. *aureus* isolates was evaluated through the disc diffusion method. Out of 543 milk samples, 310 (57.09%) were positive for SCM. Among the SCM-positive samples, *S*. *aureus* was detected in 30.32% (94/310) samples. Out of 94 isolates, 47 (50%) were determined to be multidrug resistant (MDR). Among these MDR isolates, 11 exhibited resistance to Cefoxitin, and hence were classified as methicillin-resistant *Staphylococcus aureus* (MRSA). The *S*. *aureus* isolates showed the highest resistance to Lincomycin (84.04%) followed by Ampicillin (45.74%), while the least resistance was shown to Sulfamethoxazole/Trimethoprim (3.19%) and Gentamycin (6.38%). Polymerase chain reaction (PCR) analysis revealed that 55.31% of the isolates carried *blaZ* gene, 46.80% carried *tetK* gene, 17.02% harbored the *mecA* gene, whereas, *aacA-aphD* gene was found in 13.82% samples. Our findings revealed a significant level of contamination of milk with *S*. *aureus* and half (50%) of the isolates were MDR. The isolated *S*. *aureus* harbored various antibiotic resistance genes responsible for the absorbed phenotypic resistance. The alarmingly high prevalence of MDR *S*. *aureus* isolates and MRSA strains in these cases possess a serious risk to public health, emphasizes the urgent need to address this issue to protect both human and animal health in Pakistan.

## Introduction

Mastitis is an inflammatory condition of the mammary gland and udder tissue in cows, resulting in abnormal and reduced milk yield. Typically, mastitis develops as a result of the immune system’s response to microorganisms invading the udder through the teat canal. It can also be caused due to physical damage, exposure to harmful chemicals, or high temperatures affecting the udder. It is one of the most common dairy sector diseases [1]. Mastitis may be subclinical or clinical, and can be either environmental or contagious [2]. Clinical mastitis (CM) cases include one or more of the following symptoms: irregular milk production, inflamed udder, clumps and clots in milk and systemic symptoms, including fever, fatigue, loss of appetite and depression, while the symptoms of sub-clinical mastitis (SCM) include low production of milk, and poor milk quality however the udder appears normal [3]. During SCM, in order to combat invasive pathogens, white blood cells (WBCs) such as macrophages and neutrophils move from bloodstream to the udder tissues. This results in an increased somatic cell count (SCC), and as SCC level rises, milk production declines. Milk’s protein content changes dramatically over time. Casein, the main milk protein decreases, while proteins of low nutritional value increase, negatively impacting milk [4]. Reduced milk production and discarding milk from unhealthy animals result in financial losses. Other factors contributing to losses include removing milk that contains antibiotic residues, early cow removal and replacement with expensive ones, veterinarian fees, prescription costs, extra hourly payments, and decreased commercial worth of removed cows [5].

Many contagious pathogens are responsible for mammary gland infection. Udder and teat skin of the cow are common habitats for contagious pathogens, which are commonly disseminated during the milking process from one cow to another. SCM is mostly caused by a highly contagious bacterium *Staphylococcus aureus* developing resistance and producing different virulence elements, such as endotoxins and other dangerous proteins. Mastitis in cows causes major economic losses and is associated mainly with *S*. *aureus* [1]. *S*. *aureus* is mostly found on or in the udder of cows, commonly transferred from one teat to another within a single cow or between cows while milking [6].

*S*. *aureus* contains multiple virulence factors that contribute to its toxicity. Adhesins, superantigens, capsules, and toxins are some of these factors. Adhesins are protein or glycoproteins on the bacterium’s surface that allow it to adhere to host cells. Toxins are chemicals produced by bacteria that are capable of damaging the host cells and tissues. Superantigens are poisons that can activate a high number of immune cells, resulting in an overly aggressive immunological response. Capsules are coverings that protect the bacterium from the host’s immune system [7, 8]. A variety of infections can be caused by *S*. *aureus*, including pneumonia, bone and joint infections, skin and soft tissue infections, and bloodstream infections. The way diseases develop varies depending on the type of infection. For example, in skin and soft tissue infections, bacteria might get into the body through a wound or cut, giving rise to the infection. In cases of bloodstream infections, the bacteria have the ability to enter the bloodstream and travel to other body parts, resulting in sepsis [9, 10].

In staphylococci, antibiotics primarily target nucleic acids, the cell envelope, and ribosomes to stop bacterial growth. Recent targeted drug development programs have identified many new targets, including the cell division machinery’s FtsZ and the protease ClpP [11]. The utilization of vast amounts of antibiotics for both humans and farm animals led to the *S*. *aureus* emergence, which displayed resistance to a variety of drugs. Reports show that the low- and middle-income countries are more actively contributing to the rise in antibiotic resistance rates compared to high-income countries [12, 13]. Resistance can either develop from mutations in genetic elements or via the horizontal resistance gene transfer encoded by movable genetic components like plasmids, staphylococcal cassette chromosomes, and jumping genes. In *S*. *aureus*, antibiotic resistance genes include the most prevalent tetracycline resistance genes (*tetL*, *tetO*, *tetM*, *tetK*) and erythromycin resistance genes (*ermA*, *ermC*, *ermB*). Three commonly recognized mechanisms confer tetracycline resistance to organisms: (i) Efflux by tetracycline-specific pumps, (ii) Ribosomal protection, and (iii) Enzymatic inactivation. Erythromycin resistance can arise through different mechanisms, such as efflux pumps, modification of target sites, and alteration of ribosomes [11]. *S*. *aureus* may develop multidrug resistance in one of two ways. First, multiple genes that code for drug resistance can accumulate over time within the bacteria; this accumulation often occurs on resistance plasmids. Second, increased expression of the genes that make multidrug efflux pumps that release a number of drugs may contribute to multidrug resistance [14].

*S*. *aureus* is a crucial pathogen for both humans and animals. When treating staphylococcal infections, resistance to drugs is a significant problem [15]. *S*. *aureus* exhibits significant resistance to certain antimicrobial classes, which restricts choices for therapy available to veterinary and human medicine [16]. The dairy sector and the food safety of humans are greatly affected by the multi-drug resistant (MDR) strains [17]. Multidrug resistance has been increased all over the world that is considered a public health threat. Several recent investigations reported the emergence of MDR bacterial pathogens from different origins that increase the necessity of the proper use of antibiotics. Additionally, antimicrobial susceptibility testing is routinely applied to identify the antibiotic of choice and screen for emerging MDR strains [12]. The presence of methicillin-resistant *S*. *aureus* (MRSA) in unpasteurized milk poses a major threat to public health [18]. Public health concerns have recently increased with the appearance of extremely virulent community acquired-MRSA strains [19]. The world health organization has named nosocomial infections caused by MRSA among the world’s three most challenging infectious illnesses due to its severity and prevalence [20]. The emergence of MRSA is attributed to the *mecA* resistance gene, and the presence of the *tetK* gene is recognized as a contributing factor to the development of Tetracycline resistance [14]. Gentamicin, tobramycin, and kanamycin resistance are all conferred by the *aacA-aphD* gene, which encodes an aminoglycoside modifying enzyme and *blaZ* gene causes resistance to beta-lactam antibiotics, such as amoxicillin and ampicillin [21].

*Staphylococcus aureus* exhibits an outstanding variety of virulence features, allowing it to survive in extreme host conditions and result in tissue colonization, tissue damage, and life-threatening systemic infections. The Panton-Valentine leukocidin (PVL) toxin is the main virulence factors of *S*. *aureus*, that targets WBCs. PVL enters the host plasma membrane and creates a pore or hole, it has a strong affinity for leukocytes, whereas other toxins, such as leukocidin and γ-hemolysin, are cytotoxic to WBCs and red blood cells, respectively [7]. In the context of cattle, *S*. *aureus* can induce mastitis by entering the mammary gland via the teat canal and colonizing the gland, resulting in the release of enzymes and toxins that kill host cells and tissues [22].

To ensure effective decision-making regarding the treatment of animals with antibiotics and to evade the emergence of antibiotic resistance, the pattern of *S*. *aureus*’s antibiotic resistance in raw milk needs to be monitored [23]. Therefore, the current study was designed to find out the prevalence, antibiotic resistance pattern, and the presence of antibiotic resistance genes (*mecA*, *tetK*, *aacA-aphD* and *blaZ*) in *S*. *aureus* isolated from the raw milk of cows with subclinical mastitis.

## Materials and methods

### Ethical statement

This study was approved by the Advanced Studies and Research Board (ASRB) (Dir/A&R/AWKUM/2022/9396) of the Faculty of Chemical and Life Sciences, Abdul Wali Khan University Mardan, Pakistan. The oral permissions were obtained from the farm managers regarding milk and blood collection.

### Study design

Milk samples were obtained from 543 lactating cows, including Holstein Friesian (*n* = 79), Sahiwal (*n* = 175), Cholistani (*n* = 107), and Red Sindhi (*n* = 182) from six different dairy farms across Pakistan. The samples were collected in sterilized bottles containing potassium dichromate (0.01 mg) as a preservative using aseptic techniques to avoid bacterial contamination and quickly transported in an icebox to CVS & AH AWKUM for further processing and examination of the milk contents.

### Somatic cell count (SCC)

From the milk samples, microscopic slides were prepared and the SCC was assessed using the procedure of Sharma et al. [24]. Two fields of 1 cm^2^ were made on the clean glass slide. Ten microliters of milk were added to each field and then left to air dry. After drying, xylene was added for 2–3 minutes to defat the slides, which were then fixed by adding 95% ethanol for 5 minutes and left to air dry. After fixation, 10% Giemsa stain was added to each field for 30 minutes after that, rinse with tap water. The slides were all examined through a 100X oil immersion lens of the microscope.

### Isolation and confirmation of *S*. *aureus*

*S*. *aureus* isolation and identification were performed using mannitol salt agar (MSA) plates (Oxoid Ltd., Hampshire, England) [25]. The plates were incubated for 18 to 24 hours at 37°C and were then observed for *S*. *aureus* colonies. For the isolation of pure colonies, the *S*. *aureus* colony was picked from the first culture and was sub-cultured on the same medium–MSA, then kept at 37°C for 18 to 24 hours. Further confirmation was done based on biochemical assays, including gram staining (+ coccus), catalase test (+), and coagulase test (+) [26]. As a quality-control strain, *S*. *aureus* ATCC25923 was used.

### Stock preparation

Following the manufacturer’s instructions, Luria-Bertani (LB) broth (Sigma-Aldrich Merck, Germany) was made. Fresh *S*. *aureus* colonies were added to the LB broth, and then the mixture was incubated stationary in a bacteriological incubator for 24 hours at 37°C. The broth’s turbidity after incubation showed *S*. *aureus* growth. For glycerol stock preparation, 500 μL of LB broth containing *S*. *aureus* was added to 1000 μL of 70% glycerol (Bio-Rad Inc., USA) in eppendorf and refrigerated at -40°C until further usage.

### DNA extraction

For DNA extraction of all confirmed *S*. *aureus* isolates, Chelex® 100 resin (15–50 mesh) (Bio-Rad Inc., USA) was used [27]. In a sterile falcon tube, a five percent solution of Chelex was prepared. After that, 70 μL of 5% Chelex solution was added into a sterile eppendorf through a pipette, and then a few pure colonies from a fresh culture of the bacteria were added into it via a sterile wire loop. Using a pipette, the Chelex and the bacterial colonies were mixed gently. After this, the mixture was heated in a water bath for 30 minutes at 45°C, followed by centrifugation for 10 seconds at 5000 rpm. In the new eppendorf, the supernatant was collected. The DNA was confirmed by using an agarose gel (Bio-Rad Inc., USA) of 1%.

### Molecular identification of *S*. *aureus*

All the biochemically confirmed *S*. *aureus* isolates were molecularly identified using the thermonuclease (*nuc*) gene, which is unique to this bacterium [28]. *S*. *aureus* reference strain ATCC25923 and molecular grade water were applied as respective positive and negative controls. The details of the primer (Bio-Rad Inc., USA) used is given in Table 1. For the Polymerase Chain Reaction (PCR), a total of 20 μL of reaction mixture were used [28]. DNA was initially denatured for five minutes at 94°C then 30 cycles were completed using the following thermal cycling protocol for PCR: second denaturation at 94°C for 30 seconds; annealing at 52°C for 30 seconds; extension at 72°C for 30 seconds; and final amplification for five minutes at 72°C [28]. Finally, the PCR samples were subjected to electrophoresis through a two percent agarose gel (Bio-Rad Inc., USA).

**Table 1:** Showing details of the primers used.

Gene	Nucleotide Sequence from 5’ to 3’	Annealing temperature	Base pairs	Reference
***mecA***	F: AAAATCGATGGTAAAGGTTGGC	63.6°C	532	[33]
	R: AGTTCTGCAGTACCGGATTTGC			
***tetK***	F: GTAGCGACAATAGGTAATAGT	56°C	360	[34]
	R: GTAGTGACAATAAACCTCCTA			
***aacA-***	F: GAAGTACGCAGAAGAGA	55°C	491	[34]
***aphD***	R: ACATGGCAAGCTCTAGGA			
***blaZ***	F: ACTTCAACACCTGCTGCTTTC	57°C	240	[34]
	R: TGACCACTTTTATCAGCAACC			
***nuc***	F: GCGATTGATGGTGATACGGTT	52°C	270	[28]
	R: AGCCAAGCCTTGACGAACTAAAGC			

### Antimicrobial susceptibility testing (AST)

Through the disc diffusion method, the antibiotic resistance pattern of all the *S*. *aureus* isolates was checked for nine commonly used antibiotics or antibiotic combinations across seven different antibiotic classes, namely Penicillins (Amoxicillin (10 μg), Ampicillin (10 μg)), Aminoglycosides (Amikacin (30 μg), Gentamycin (10 μg)), Tetracyclines (Tetracycline (30 μg)), Fluoroquinolones (Levofloxacin (5 μg)), Cephamycins (Cefoxitin (30 μg)), Lincosamides (Lincomycin (10 μg)) and Sulfonamides (Sulfamethoxazole/ Trimethoprim (25 μg)) (ThermoFisher Scientific, USA). To evaluate AST, the *S*. *aureus* ATCC25923 was employed as a quality control strain. According to the CLSI guidelines disk diffusion test was performed [29]. MRSA Phenotypic identification was based on resistance to Cefoxitin [30–32].

### Detection of antibiotic resistance genes through PCR

Antibiotic resistance genes, *mecA*, *tetK*, *blaZ*, and *aacA-aphD*were detected and amplified using conventional PCR. From our lab, *S*. *aureus* strains positive for *mecA*, *tetK*, *blaZ*, and *aacA-aphD* genes as a positive control, and molecular grade water as a negative control was used [27]. The details of the primers used are given in Table 1. For the PCR, a total of 20 μL of reaction mixture was used. DNA was initially denatured for 10 minutes at 95°C, then 35 cycles were completed using the following thermal cycling protocol for PCR: second denaturation at 95°C for 30 seconds; annealing at 56°C, 63.6°C, 55°C, and 57°C for 30 seconds for *tetK*, *mecA*, *aacA-aphD*, and *blaZ* respectively; and extension at 72°C for 30 seconds. During the final amplification phase, at 72°C, the complete single DNA strand was polymerized for 10 minutes [33, 34]. The desired genes were verified using an agarose gel of 1.5 percent.

### Statistical analysis

Data was collected from well-known dairy farms and inserted into Excel sheets. Statistical analysis was done to determine relationships between different breeds and farms of cattle with the prevalence of *S*. *aureus*. P < 0.05 was considered significant when using the chi-square test. Pearson’s Correlation Coefficient was determined to measure the relationship between the identified resistance genes and the tested antibiotic classes. The SPSS version 22 was used to analyze the data.

## Results

Mastitis is an infectious condition that affects the mammary gland and occurs as a result of injury with the aim of neutralizing or killing the infectious agents and preparing the path for healing and the restoration to normal function. Mastitis may be subclinical or clinical and depending on the major reservoir and transmission methods, can be either environmental or contagious. Mastitis may have a number of etiological causes; however, bacteria are most commonly to blame. *S*. *aureus* is among the most common bacteria causing mastitis. *S*. *aureus* is making the infection more and more challenging, because with the passage of time it evolves from being mono-drug resistant to multi-drug resistant e.g MRSA. In this study 543 bovine milk samples were collected from different dairy farms in Pakistan. Subclinical mastitis was determined in the samples by evaluating the somatic cell count. Then subclinical bovine mastitis associated *S*. *aureus* were isolated and studied. The prevalence of *S*. *aureus* in SCM was assessed, the isolates antibiotic susceptibility was checked and antibiotic resistance genes in these isolates were determined.

### Detection of sub-clinical mastitis

Depending on the quantity of somatic cells in one ml of milk, the health status of cows was determined. The SCC was done for all 543 samples. Cows were considered to have SCM if the SCC exceeded 200,000 cells/ml in milk. Out of 543 total milk samples, 57.09% (n = 310) had subclinical mastitis, of which 104 were Red Sindhi, 100 were Sahiwal, 61 were Cholistani, and 45 were Holstein Friesian (Table 2).

**Table 2:** Showing SCC in different breeds of cows.

Groups	Red Sindhi (n = 182)	Sahiwal (n = 175)	Cholistani (n = 107)	Holstein Friesian (n = 79)	Total (n = 543)
Group-A	78 (33.48%)	75 (32.19%)	46 (19.74%)	34 (14.59%)	233
Group-B	104 (33.54%)	100 (32.28%)	61 (19.67%)	45 (14.51%)	310

Group -A. Healthy cows having SCC ≤200,000 cells/ml of milk

Group -B. Subclinical mastitic cows having SCC > 200,000 cells per ml of milk.

### Phenotypic characteristics of the recovered *S*. *aureus* isolates

The *S*. *aureus* on MSA showed strong growth with pen pointed yellow colonies, displaying mannitol fermentation. The colonies revealed a round and well-defined morphology, differentiating them from other bacteria on MSA. Gram staining of *S*. *aureus* revealed Gram-positive cocci. The retrieved *S*. *aureus* isolates were poisitive for catalase and coagulase tests.

### Prevalence of *S*. *aureus* in SCM positive samples

A total of 310 milk samples positive for SCM were examined for *S*. *aureus* presence. *S*. *aureus* was detected in 30.32% (94/310) milk samples. *S*. *aureus* was confirmed by its phenotypic characteristics and was identified molecularly through the species-specific *nuc* gene.

### Dairy farms wise and breed wise prevalence of *S*. *aureus*

**Fig 1:**
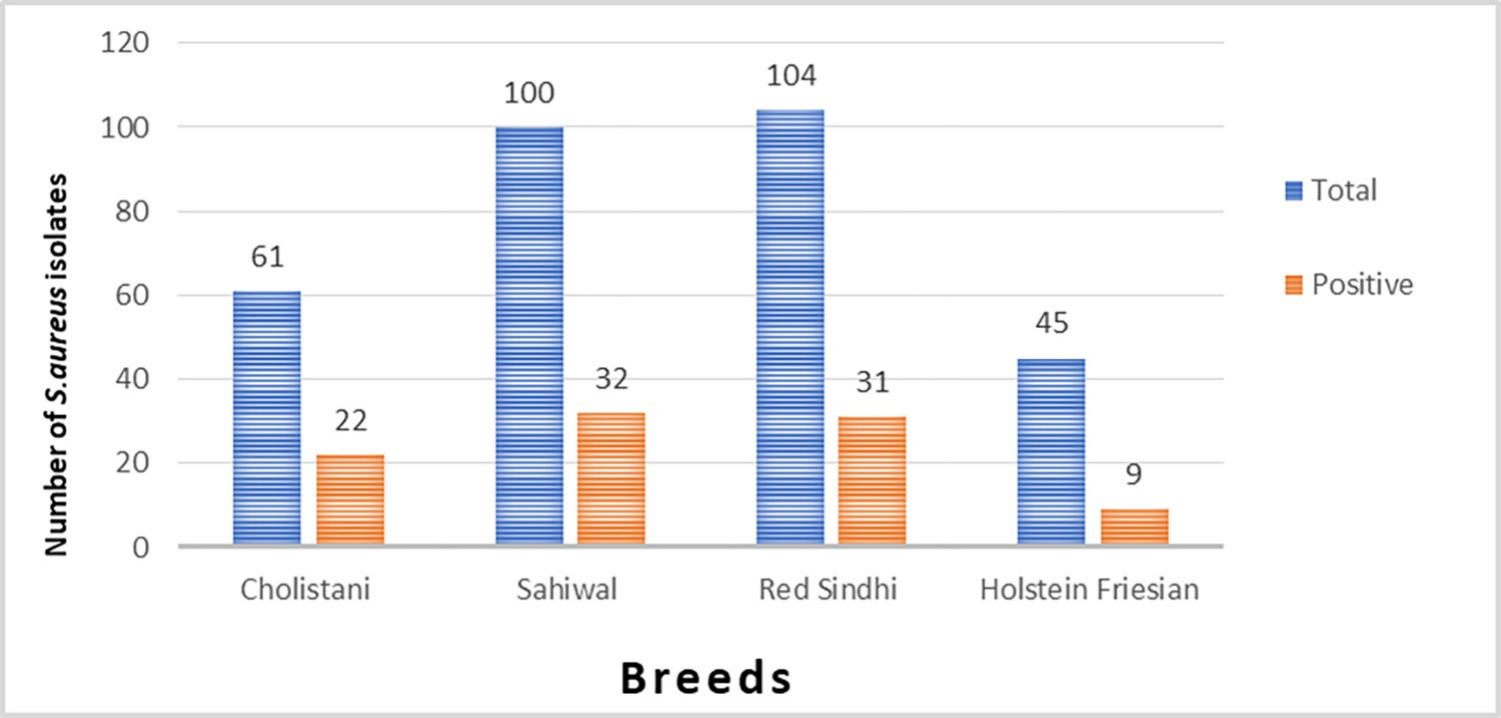
*S*. *aureus* prevalence by breed in SCM milk samples.

The highest *S*. *aureus* prevalence was noted in Red Sindhi cattle farm Hub (39.13%), followed by Govt. livestock farm Jugaitpeer Cholistan (36.06%), Livestock experimental station Bhadurnagar Okara (32%) and Local Red Sindhi Gharo (28.78%). The lowest prevalence was recorded in Livestock experimental station Korangi Karachi and Govt. cattle breeding and dairy farm Harichand (20%). The p-value was more than 0.05; thus, the prevalence difference between dairy farms is considered nonsignificant (Table 3). In breed wise prevalence, the Cholistani breed had the greatest rate of *S*. *aureus* (36.06%, n = 22/61), followed by Sahiwal (32%, n = 32/100) and Red Sindhi (29.80%, n = 31/104). In comparison, the lowest prevalence was noted in Holstein Friesian (20%, n = 9/45). Differences in breed-wise prevalence are nonsignificant (p = 0.591) (Fig 1).

**Table 3:** Farms-wise prevalence of *S*. *aureus*.

Dairy Farms (Cow Breed)	Total	SCM positive	*S*. *aureus* positive in SCM	*S*. *aureus* prevalence in SCM	p-value
Red Sindhi Cattle Farm Hub (Red Sindhi)	40	23	9	39.13%	P = 0.73^NS^
Govt. Livestock Farm Jugaitpeer Cholistan (Cholistani)	107	61	22	36.06%	
Livestock Experimental Station Bhadurnagar Okara (Sahiwal)	175	100	32	32%	
Local Red Sindhi Gharo (Red Sindhi)	116	66	19	28.78%	
Livestock Experimental Station Korangi Karachi (Red Sindhi)	26	15	3	20%	
Govt. Cattle Breeding & Dairy Farm Harichand (Holstein Friesian)	79	45	9	20%	

^NS^ Not significant

### Antibiotic susceptibility testing

AST was performed on all 94 isolates of *S*. *aureus*. According to the CLSI guidelines, all verified *S*. *aureus* isolates were divided into three categories: resistant, intermediate, and susceptible for all antibiotics tested. Out of 94 isolates, 88% exhibited resistance to at least one of the antibiotics, including Amoxicillin, Gentamycin, Amikacin, Tetracycline, Levofloxacin, Cefoxitin, Lincomycin and Sulfamethoxazole/Trimethoprim. The isolates of *S*. *aureus* exhibited the highest levels of Lincomycin resistance (84.04%), followed by Ampicillin (45.74%), Amoxicillin (42.5%), and Tetracycline (17.02%), while the least resistance was shown to Sulfamethoxazole/ Trimethoprim (3.19%) and Gentamycin (6.38%) (Table 4).

**Table 4:** Antibiogram of *S*. *aureus* isolates.

Antibiotic class	Antibiotic	Code	Quantity ( μg)	*S*. *aureus* antibiotic resistance pattern (n = 94)		
				Resistant (%)	Intermediate (%)	Sensitive (%)
Penicillins	Amoxicillin	AML	10	40 (42.5)	1 (1.12)	53 (56.38)
	Ampicillin	AMP	10	43 (45.74)	9 (9.58)	42 (44.68)
Aminoglycosides	Gentamycin	CN	10	6 (6.38)	4 (4.26)	84 (89.36)
	Amikacin	AK	30	7 (7.44)	26 (27.67)	61 (64.89)
Tetracyclines	Tetracycline	TE	30	16 (17.02)	22 (23.41)	56 (59.57)
Fluoroquinolones	Levofloxacin	LEV	5	7 (7.44)	7 (7.46)	80 (85.10)
Cephamycins	Cefoxitin	FOX	30	11 (11.7)	2 (2.13)	81 (86.17)
Lincosamides	Lincomycin	MY	10	79 (84.04)	12 (12.77)	3 (3.19)
Sulfonamides	Sulfamethoxazole/ Trimethoprim	SXT	25	3 (3.19)	1 (1.07)	90 (95.74)

### Detection of antibiotic resistance genes

Through PCR, *mecA*, *tetK*, *aacA-aphD and blaZ* resistance genes were checked in all *S*. *aureus* isolates. Out of 94 samples, the *blaZ* gene was discovered in 52 samples, indicating its relatively widespread presence. In comparison, *tetK* gene was found in 44 samples, showing a lower frequency than *blaZ*. The *mecA* gene appeared in 16 samples whereas, *aacA-aphD* was found in just 13 samples.

The Pearson’s Correlation Coefficient between the identified resistance genes and the tested antibiotic classes was estimated in the overall *S*. *aureus* isolates. A positive correlation (r = 0.54) was found between the presence of *blaZ* gene and Penicillins resistance, between the presence of *tetK* gene and Tetracyclines resistance (r = 0.75), and between the presence of *mecA* gene and Cephamycins resistance (r = 0.72). The correlation between the *aacA-aphD* gene and Aminoglycosides resistance was r = 0.32.

### MDR *S*. *aureus* isolates

A total of 94 *S*. *aureus* isolates were tested for antibiotic resistance phenotypically, in which 47 isolates were determined to be MDR. Isolates were classified as MDR based on the criteria outlined by [35]. Among these MDR isolates, 23.40% (11 samples) exhibited resistance to Cefoxitin, and hence were classified as MRSA. Notably, 97.87% MDR isolates were non-susceptible to Lincomycin, with significant rates of non-susceptibility observed for Ampicillin (74.46%), Amoxicillin (72.34%), Tetracycline (63.82%) and Amikacin (25.53%). In contrast, only 6.38% of MDR isolates were non-susceptible to Sulfamethoxazole/ Trimethoprim, while 14.89% each were non-susceptible to Gentamycin and Levofloxacin. As shown in Table 5, 91.48% of MDR *S*. *aureus* isolates carried the *blaZ* gene, while 72.34% had the *tetK* gene, 29.74% had the *mecA* gene, and 23.40% carried the *aacA-aphD* gene.

**Table 5:** Antibiotic resistance pattern of the MDR *S*. *aureus* isolate and the expressed resistance genes.

Isolate ID	Antibiotics	Expressed Resistance Genes	Isolate ID	Antibiotics	Expressed Resistance Genes
SA4	MY, AMP, AML, TE	*tetK*, *bla*Z, aacA-aphD	SA55	MY, AML, AMP, TE	*tetK*, *blaZ*,
SA5^a^	MY, FOX, AML,	*tetK*, *blaZ*, *MecA*	SA57	MY, AML, AMP, CN	*blaZ*, aacA-aphD
SA8	MY, AML, AMP, TE	*tetK*, *blaZ*, *MecA*	SA58 ^a^	MY, FOX, AML, AMP, TE, AK	*tetK*, *blaZ*, *MecA*
SA9 ^a^	MY, FOX, AMP, TE	*tetK*, *blaZ*, *MecA*	SA59 ^a^	MY, FOX, AML, TE	*tetK*, *blaZ*, *MecA*
SA10	MY, AML, AMP, SXT, CN	*tetK*, *blaZ*, aacA-aphD	SA63	MY, AML, AMP, TE	*tetK*, *blaZ*,
SA14	MY, AML, AMP, AK, TE	*tetK*, *blaZ*,	SA67	MY, AML, AMP, AK, TE	*tetK*, *blaZ*, aacA-aphD
SA16	MY, AMP, TE	*tetK*, *blaZ*	SA68	MY, AML, AMP, SXT,	*tetK*, *blaZ*,
SA22	MY, AMP, AK	*blaZ*, *MecA*	SA70	MY, AMP, CN, AK	aacA-aphD
SA23	MY, AML, AMP, TE,	*tetK*, *blaZ*, *MecA*	SA72	MY, AMP, LEV, TE	*tetK*, *blaZ*,
SA25	MY, AMP, AK	*blaZ*	SA73	MY, AML, AMP, LEV	*tetK*, *blaZ*,
SA27^a^	MY, FOX, AML, AMP, TE	*tetK*, *blaZ*, *MecA*	SA76	MY, AML, LEV	*blaZ*,
SA28	MY, AML, AMP, TE	*tetK*, *blaZ*,	SA78 ^a^	MY, FOX, AML, TE	*tetK*, *blaZ*,
SA29 ^a^	MY, FOX, AML, AMP	*blaZ*, *MecA*	SA81	MY, AML, LEV	*blaZ*,
SA30	AMP, TE, CN	*tetK*, *blaZ*,	SA82 ^a^	MY, FOX, AML, AK, TE	*tetK*, *blaZ*, *MecA*, aacA-aphD
SA36 ^a^	MY, FOX, AML, TE	*tetK*, *blaZ*, *MecA*	SA83	MY, AMP, LEV, TE	*tetK*, *blaZ*,
SA39 ^a^	MY, FOX, AK	*MecA*	SA84	MY, AML, AMP, SXT	*blaZ*,
SA40	MY, AML, AMP, LEV	*tetK*, *blaZ*,	SA85	MY, AML, AMP, TE	*tetK*, *blaZ*,
SA42	MY, AML, AMP, TE	*tetK*, *blaZ*,	SA87	MY, AML, AMP, TE	*tetK*, *blaZ*,
SA43	MY, AML, AMP, CN,	*MecA*, aacA-aphD	SA89	MY, AML, AMP, TE	*tetK*, *blaZ*,
SA45	MY, AML, AMP, AK, TE	*tetK*, *blaZ*, aacA-aphD	SA90	MY, AML, AK,	*blaZ*, aacA-aphD
SA49	MY, AMP, TE, CN	*tetK*, *blaZ*,	SA91^a^	MY, FOX, AK, TE	*tetK*, *MecA*, aacA-aphD
SA51	MY, AML, TE	*tetK*, *blaZ*,	SA92	MY, AML, TE	*tetK*, *blaZ*,
SA52	MY, AMP, TE, AK	*tetK*, *blaZ*, aacA-aphD		MY, AMP, LEV, TE	*blaZ*,
SA53	MY, AML, AMP, CN	*blaZ*,			

MY (Lincomycin), AMP (Ampicillin), TE (Tetracycline), FOX (Cefoxitin), AML (Amoxicillin), SXT (Sulfamethoxazole/ Trimethoprim), AK (Amikacin), CN (Gentamycin), LEV(Levofloxacin)

^**a**^ Methicillin-resistant *Staphylococcus aureus* (MRSA) strains

The Pearson’s Correlation Coefficient analysis in the MDR isolates reveals a positive correlation between the resistant phenotypes and the resistance genes. The correlation between the Penicillin’s resistance and the presence of the *blaZ* gene was r = 0.69. Cefoxitin resistance was positively correlated (r = 0.73) with the presence of the *mecA* gene. Similarly, Tetracyclines resistance was positively correlated (r = 0.72) with the presence of *tetK* gene. The correlation between the Aminoglycosides resistance and the presence of *aacA-aphD* gene was r = 0.59.

## Discussion

In this study, out of 543 total milk samples, 57.09% (n = 310) had SCC levels above 200,000, indicating SCM. In Pakistan, Najeeb et al. [36] reported a 39.2% SCM prevalence, 80.8% of the total cases were caused by *S*. *aureus*, and 18.8% of milk samples contained MRSA based on AST and 6.5% based on the molecular study. A review article authored by Bari et al. [37] discovered that SCM varied in cows, from 18% to 87% in India. Chen et al. [38] published a study in 2022 indicated that in seven regions of China, the estimated prevalence of subclinical mastitis ranged between 36.4% and 50.2%. Similar research was done by Tesfaye et al. [39] and found a comparatively higher prevalence of SCM, i.e., 69.4%. A previous study from Muzaffar Garh Pakistan, reported that 45% of crossbred cows had SCM [40]. In South Ethiopia’s Hawassa milk shed, dairy cattle herds were the subject of a study by Abebe et al. [41] they reported a 59.2% prevalence of SCM. Sefinew et al. [42] reported 9.7% and 37.2% prevalence of CM and SCM, respectively in milk collected from local zebu breeds and zebu-Holsteins cross breeds in Ethiopia.

Breed-wisely, SCM was found to be most prevalent in Sahiwal (57.14%) and Red Sindhi (57.14%), followed by Cholistani (57%) and Holstein Friesian (56.96%). A research by Bachaya et al. [43], found that SCM is common in crossbred cows (36%) in Muzaffar Garh, Pakistan. Dar et al. [25] reported a prevalence of 74.61% and 25.38% of SCM in crossbred and native cows, respectively.

*S*. *aureus* was prevalent in this study at a rate of 30.32 percent (n = 94 / 310). Compared to this, the prevalence in other countries was recorded as 43.1%, 46.6%, 50.0%, and 30.6% in China, USA, Poland, and Ethiopia, respectively [39, 44–46]. A higher occurrence of *S*. *aureus* in SCM milk samples was recorded by Ren et al. [47] from southern Xinjiang China which was 77.38%, while the prevalence of *S*. *aureus* in Beijing reported by Wang et al. [48] was 46.2%. On the other hand, from two different regions of Ethiopia, 42.85% and 51.2% prevalence rates of *S*. *aureus* were reported by Bude and Mengesha [3] and Abebe et al. [49], respectively. The *S*. *aureus* prevalence in the previous studies and current study can vary due to several reasons, such as study design, sampling method, population characteristics, and geographic location [50].

The bacteria become resistant to a drug by one of these mechanisms; inactivating or modifying the drug enzymatically, modifying the binding site of the drug, drug efflux, protecting the target by displacing the drug, circumvent mechanisms involving gaining of new drug resistant targets [11]. In the current research, 47 (50%) of the 94 *S*. *aureus* isolates were MDR. A previous study from Pakistan reported nearly the same prevalence (44.44%) of MDR bacteria in the milk samples [36]. A comparable MDR prevalence (46.8%) was observed in another study carried out in China [44]. The current study’s MDR prevalence was significantly greater than reports from other countries, such as Indonesia (10%), Greece (13.3%), and Kenya (29.67%) [51–53]. The isolates of *S*. *aureus* exhibited the highest levels of Lincomycin resistance (84.04%), followed by Ampicillin (45.74%), Amoxicillin (42.5%), and Tetracycline (17.02%). This high resistance rate is because of MDR strains’ emergence, escalated by the excessive misuse of antibiotics in veterinary practice. In contrast, the isolates showed the highest susceptibility to Sulfamethoxazole/ Trimethoprim (95.74%) and Gentamycin (89.36%). The isolates’ susceptibility to Sulfamethoxazole/Trimethoprim is attributed to the synergistic effect of the drug, while Gentamycin is used less frequently, resulting in lower resistance towards it [54]. The high prevalence of MDR *S*. *aureus* strains among SCM cases indicates the need for a more vigilant approach toward the use of antibiotics in veterinary practices. Additionally, it is advised to periodically test the antimicrobial sensitivity of the pathogens before usage and monitor the rational use of medications.

The *mecA*, *tetK*, *aacA-aphD* and *blaZ* resistance genes were checked in all *S*. *aureus* isolates. The *blaZ* gene was found in 52 (55%) samples, indicating its relatively high prevalence. Whereas, 44 (46.80%) of *S*. *aureus* isolates had the *tetK* gene, the *mecA* gene was found in 16 (17.02%) isolates, and *aacA-aphD* was found in just 13 (13.82) samples. The two main mechanisms *S*. *aureus* employs to develop β-lactam resistance are the penicillinase production and penicillin-binding protein modification [55]. *S*. *aureus* becomes resistant to tetracycline by activating the efflux pump encoded by the *tetK* gene [19]. Gentamicin and Amikacin belong to the aminoglycoside class of antibiotics, and transferrable plasmids that encode bifunctional aminoglycoside-modifying enzymes like *aacA-aphD* can cause resistance to them [56]. Similarly, Amoxicillin and Ampicillin, which are both penicillin-class antibiotics, are not any more effective compared to other antibiotics against bacteria that make beta-lactamase enzymes such as *blaZ* [57]. An earlier investigation from China carried out by Qu et al. [34] reported nearly the same occurrence of *mecA* (16%) and *tetK* gene (31%), whereas, a relatively higher frequency of *aacA-aphD* (23%) and *blaZ* gene (95%) in *S*. *aureus* isolates. Liu et al. [58] discovered that 12.90% of the *S*. *aureus* isolates carried the *mecA* gene in large-scale dairy farms in China. In contrast, Mbindyo et al. [52] from Kenya found a higher prevalence of *mecA* (25%) and a lower prevalence of *tetK* (0%) and *blaZ* gene (41%) in the *S*. *aureus* isolates.

In this study, there is discrepancy between the presence of antibiotic resistance genes and the frequency of resistance in the phenotype. It is common for bacteria to have an antibiotic resistance gene but not being resistant to the concerned drug. This is due to the fact that the presence of an antibiotic resistance gene does not guarantee that the bacteria will be resistant to the drug. Additional factors such as the presence of other genes, expression of the gene, and environmental conditions can all affect whether or not the bacteria will develop antibiotic resistance [59].

In the current research the raw milk is significantly contaminated with *S*. *aureus* including the MDR-MRSA strains that carried the antibiotic resistance genes. This resistance can be transmitted from cows to humans through the consumption of contaminated milk or contact with infected animals, directly endangering human health [6]. Furthermore, the persistence of antibiotic-resistant bacteria in the agricultural setting, such as on dairy farms, can facilitate the spread of resistance genes throughout the wider ecosystem, worsening the global antibiotic resistance crisis.

## Conclusions

It is concluded that the raw milk of the studied breeds of cows is significantly contaminated with antibiotic resistant *S*. *aureus* including the MDR isolates and MRSA strains. The isolated *S*. *aureus* harbored various resistance genes responsible for the absorbed phenotypic resistance. The alarmingly high prevalence of MDR *S*. *aureus* isolates and MRSA strains in these cases present a serious risk to public health. Therefore, this study has provided valuable information that may be helpful to reduce public health risks associated with acquisition of MDR-MRSA along with the milk value chain. Consequently, we recommend continuous surveillance and monitoring of antibiotic resistant *S*. *aureus*, including the MDR-MRSA to reduce the emergence and spread of the milk-borne drug-resistant *S*. *aureus* strains, to protect the public health and promote sustainable one health is Pakistan.
